# Confirmatory factor analysis and examination of the psychometric properties of the eating beliefs questionnaire

**DOI:** 10.1186/s12888-017-1394-z

**Published:** 2017-07-03

**Authors:** Amy L. Burton, Phillipa Hay, Sabina Kleitman, Evelyn Smith, Jayanthi Raman, Jessica Swinbourne, Stephen W. Touyz, Maree J. Abbott

**Affiliations:** 1 School of Psychology, University of Sydney, Sydney, NSW Australia; 2 School of Medicine, Western Sydney University, Sydney, NSW Australia; 3 Translational Health Research Institute (THRI), School of Medicine, Western Sydney University, Sydney, NSW Australia; 4 School of Social Sciences and Psychology, Western Sydney University, Sydney, NSW Australia; 5 The Boden Institute of Obesity, Nutrition, Exercise and Eating Disorders, Sydney, NSW Australia

**Keywords:** Eating disorders, Binge eating, Cognitive, Beliefs, Self-report, Questionnaire, Factor analysis, Psychometric, Validity, Reliability

## Abstract

**Background:**

The Eating Beliefs Questionnaire (EBQ) is a 27-item self-report measure that assesses positive and negative beliefs about binge eating. It has been validated and its factor structure explored in a non-clinical sample. This study tested the psychometric properties of the EBQ in a clinical and a non-clinical sample.

**Method:**

A sample of 769 participants (573 participants recruited from the university and general community, 76 seeking treatment for an eating disorder and 120 participating in obesity research) completed a battery of questionnaires. A subset of clinical participants with a diagnosis of Bulimia Nervosa or Binge Eating Disorder completed the test-battery before and after receiving a psychological treatment (*n* = 27) or after allocation to a wait-list period (*n* = 28), and a subset of 35 community participants completed the test battery again after an interval of two-weeks. Confirmatory Factor Analysis (CFA) was performed.

**Results:**

CFA found a two-factor structure that provided a good fit to the data, supporting the solution presented in the development paper. Items with poor psychometric properties were removed, resulting in a 16 item measure. EBQ scores were found to correlate with binge eating episode frequency, increases in body mass index (BMI), and measures of eating disorder behaviours and related psychopathology. The EBQ was found to have excellent internal consistency (α = .94), good test-retest reliability (*r* = .91) and sensitivity to treatment.

**Conclusion:**

These findings indicate that the EBQ is a psychometrically sound and clinically useful measure.

## Background

### Binge eating

Binge eating, a core feature of eating disorder presentations, is defined as eating a large quantity of food in a discrete period of time, coupled with a sense of loss of control over one’s eating [1]. Binge eating occurs along a continuum from normal to disordered eating [2]. As such, this behaviour not only occurs in cases of Bulimia Nervosa (BN), Anorexia Nervosa (AN; binge/purge subtype), Binge Eating Disorder (BED), and other atypical eating disorders [1], but is also present at a sub-threshold level among the general community [2]. Binge eating has been found to be associated with significant mental and physical health problems, as well as impaired quality of life and social functioning [3].

BED is the most common eating disorder with a reported prevalence of 1.9% internationally [4] and 2.3% in Australia [5]. Like all eating disorders, BED predominately affects females, however it has been estimated that 30–40% of BED sufferers are male [4, 5]. However, estimates in the Australian population suggest that up to 7.2% Australians are reported to be engaging in binge eating episodes; a rate that is increasing [5]. Despite the high prevalence and substantial physical and emotional impact of binge eating, there remains uncertainty in the literature regarding the underlying mechanisms that act to maintain binge eating behaviour. There is also no consensus in the literature regarding whether there are significant differences in key maintaining factors (such as beliefs about food and eating) between sub-threshold and clinically significant levels of binge eating. Additionally, there is a lack of understanding of whether these factors are different depending on the diagnostic group [6]. A clear understanding of the maintaining factors underlying binge eating behaviour is critical for the development of effective treatment models. This is of particular importance given the high rates of binge eating and the significant impact that this behaviour has on afflicted individuals.

Currently, many of the more influential cognitive models of binge eating focus on the role of restricting/dieting behaviour, low self-esteem, and preoccupations with body, shape and weight. Few consider core cognitive processes that contribute to the maintenance of the binge eating behaviour – an important consideration given the very aversive and unpleasant nature of binge eating [7]. However, Cooper, Wells and Todd’s model [8] emphasises the role of certain cognitions in maintaining binge eating behaviours. In their model, the maintenance processes are presented as a cycle of behaviours that are driven by core beliefs and related sets of metacognitive beliefs. Metacognition refers to thoughts about cognition; this consists of thoughts and beliefs related to the monitoring, control, interpretation and appraisal of cognitive events and behaviours [9]. Cooper et al. [8] categorised these sets of beliefs as either *positive* thoughts about the role of binge eating in reducing emotional distress, *permissive* thoughts which allow the individual to engage in a binge, and thoughts of ‘no control’ or *negative beliefs.* Specifically, the model suggests that the initial trigger of a binge eating episode is a distressing event which leads the individual to experience negative thoughts about themselves, e.g., “I’m a failure”, which is accompanied by feelings of anxiety, depression or guilt. The model proposes that these individuals hold positive beliefs about eating, e.g., “eating helps me to cope”, and permissive beliefs about binge eating, e.g., “it’s okay to binge on something nice”, and they commence a binge eating episode as a way to cope with these emotions. Initially, the eating is effective in reducing the intensity of their distress, and thereby further reinforces these positive beliefs about eating. The model also emphasises the importance of the negative beliefs related to ‘no control’ over eating, e.g., “once I start eating I can’t stop”, in the commencement and maintenance of binge eating behaviour. When these negative beliefs are activated, the individual feels helpless and out of control, thereby further increasing emotional distress, which in-turn, increases binge eating behaviour due to the positive beliefs and the distress regulation effect. The model therefore suggests that it is the combination of these two types of metacognitive beliefs, positive and negative, which serve to maintain the vicious cycle of binge eating behaviour. Positive and negative beliefs as described by Cooper et al. form the basis of the items in the Eating Beliefs Questionnaire [10], which is the focus of the present study.

### Measures of binge eating

Currently, most of the available measures used to screen for BED focus on behaviours and diagnostic criteria; some examples include the Eating Disorders Examination Questionnaire (EDE-Q) [11], Eating Disorders Diagnostic Scale (EDDS) [12], Eating Attitudes Test (EAT-26) [13], and the Binge Eating Scale (BES) [14]. Other measures of beliefs typically focus on beliefs more relevant to AN or BN patients, such as beliefs that relate to the drive for thinness and the fear of fatness, for example, the Eating Disorders Beliefs Questionnaire (EDBQ) [15], and Three Factor Eating Questionnaire (TFEQ) [16]. Very few available measures focus on beliefs that are relevant to BED; two such exceptions are the Dutch Eating Behaviour Questionnaire (DEBQ) [17] and the Thoughts Questionnaire [18].

A recent systematic review [19] of the psychometric properties of published self-report measures assessing binge eating behaviour and psychopathology concluded that none of the 29 included measures had evidence demonstrating that they met criteria for adequate psychometric properties across all nine categories assessed (content validity, construct validity, internal consistency, criterion validity, reproducibility: agreement, reproducibility: reliability, responsiveness, floor and ceiling effects, and interpretability), using best practice assessment of psychometric properties [20]. The two measures identified in the systematic review to have the most evidence for their psychometric properties (evidence for adequacy for six out of nine of the properties assessed), were the Bulimia Test Revised (BULIT-R) [21] and the Bulimic Investigatory Test Edinburgh (BITE) [22]. Both the BULIT-R and the BITE were specifically tailored to assess binge eating symptomatology, serving the purpose of screening for bulimic symptoms and their severity, however these measures do not assess beliefs held by the individual about the process of binge eating. Of the few cognitive measures assessed in this review, the EDBQ [15] had the most evidence for possessing good psychometric properties (demonstrating adequacy for five out of the nine properties assessed). The EDBQ assesses certain cognitions thought to be relevant to eating disorders, including negative self-beliefs, beliefs related to acceptance by self/others, and control over eating. However, the EDBQ does not assess other areas of cognition believed to be important in maintaining binge eating such as positive, negative or permissive beliefs about food and eating. As such, there is a gap in the literature in terms of the availability of a psychometrically-sound self-report measure that examines thoughts or cognitions that are specifically relevant to binge eating.

### Eating beliefs questionnaire development

Based on the work of Cooper et al. [8], Groves, Baillie and Abbott [10] designed the Eating Beliefs Questionnaire (EBQ), a self-report assessment tool that measures beliefs about food, eating and bingeing that are believed to play a key role in maintaining binge eating behaviour. The 27-item questionnaire measures both *positive* and *negative* beliefs about binge eating. Positive beliefs are characterised as beliefs that binge eating will assist in alleviating negative affect, while negative beliefs relate to cognitions that Cooper et al. [8] referred to as “no control” beliefs. That is, negative beliefs relate to thoughts that one has no control over binge eating behaviour in terms of a perceived inability to resist urges to binge eat and an inability to stop eating once an episode of binge eating has started. Results of an exploratory factor analysis in a non-clinical sample identified a two-factor structure – negative and positive beliefs [10]. The EBQ was also found to demonstrate evidence that it is a valid and reliable measure of eating-related beliefs [10]. The EBQ scores correlated significantly with Body Mass Index (BMI) as well as with a measure of depression, anxiety and stress – factors which are known to be correlate with binge eating [3, 4, 10]. Although demonstrating promising results in the initial validation study, additional research is warranted to further investigate the psychometric properties and clinical utility of this measure. The structure of the current EBQ has not yet been validated with a confirmatory factor analysis, nor has it been validated with a clinical sample.

## Aims and objectives

The present study aimed to validate the factor structure and psychometric properties of the Eating Beliefs Questionnaire (EBQ) [10], using a clinical and non-clinical sample. This study also aimed to provide a thorough assessment of the validity and reliability of the EBQ, as well as investigate additional psychometric properties of this measure, including treatment sensitivity.

## Hypotheses

We hypothesised that the confirmatory factor analysis would support the two factor solution identified in the initial exploratory factor analysis conducted by Groves et al. [10] with separate distinct factors for items assessing positive and negative beliefs about eating. We also expected that the EBQ scores would demonstrate convergent validity by being positively correlated with binge eating symptoms, BMI, and measures of mood/affect, eating disorder psychopathology, negative self-beliefs and poor distress tolerance. We expected the strength of these associations to be greater for constructs thought to be more relevant to binge eating (e.g., poor distress tolerance and poor self-esteem) than for constructs more relevant for the restrictive eating disorders (e.g., restraint and high standards for self). It was also predicted that the EBQ would demonstrate adequate internal consistency, test-retest reliability over 2 to 10 weeks, and sensitivity to treatment following a psychological intervention.

## Methods

### Participants

A total of 769 participants completed this study (67.9% female, mean age = 27.99 years, SD = 12.17 years, range = 17 to 72 years, mean BMI = 26.25, SD = 8.08). Of these, 290 were recruited from the general community (67.9% female, mean age = 27.54 years, SD = 9.57 years, mean BMI = 24.42, SD = 6.70), 283 were recruited from a sample of first year psychology students from The University of Sydney (52.3% female, mean age = 20.23, SD = 4.8 years, mean BMI = 22.58, SD = 4.12), 76 were recruited as part of studies assessing new treatments for binge eating (100% female, mean age = 35.97 years, SD = 17.68, mean BMI = 28.01, SD = 7.23), and 120 were recruited as part of a study on obesity (58% female, mean age = 42.31 years, SD = 9.51 years, mean BMI = 38.22, SD = 7.23).

### Design

Dependent variables were participants’ scores on the various measures, including the EBQ, binge eating severity (as determined by a semi-structured diagnostic interview, the EDE, or its companion self-report questionnaire- the EDE-Q), and BMI.

### Measures

For the purpose of assessing the EBQ’s construct validity and relevance to binge eating, the test battery consisted of measures that are known to correlate with binge eating and/or eating disorder severity [3, 4, 8, 10, 19]. Together, these measures assessed body mass (BMI), overall mood and distress (Depression Anxiety Stress Scales; DASS-21), eating disorder symptoms and related behaviours (EDE-Q and DEBQ), eating disorder related cognitions (EDE-Q and EDBQ), negative core-beliefs (Eating Disorders Core Beliefs Questionnaire; ED-CBQ) and poor distress tolerance (Difficulty in Emotion Regulation Scale; DERS).

*The Eating Beliefs Questionnaire (EBQ)* [10]. The EBQ is a 27 item self-report metacognitive measure, consisting of two subscales that assesses positive and negative thoughts about eating and urges to eat when not hungry. An example of a positive item is “Eating helps me to feel calm”, and an example of a negative item is “I have no willpower in relation to food”. Participants rate how much they agree with each of the items from 1 (strongly disagree) to 5 (strongly agree).

*Body Mass Index (BMI)*. The height and weight of participants was recorded so that BMI could be determined. BMI is calculated by dividing an individual’s weight in kilograms by their height in metres squared (BMI = kg/m^2^). BMI categories indicate if an individual’s body weight is within a healthy range. Individuals with BMIs above and below the healthy weight range are at greater risk of diseases such as cardio-vascular disease and diabetes [23].

*The Eating Disorder Examination* (EDE) [7]. The EDE is a clinician-administered semi-structured interview which assesses eating disorder symptoms and associated features over the previous 28 days. The EDE provides a global score as well as four subscale scores: dietary restraint, eating concern, weight concern and shape concern. Examples of items include: “Have you been deliberately trying to limit the amount of food you eat to influence your shape and weight (whether or not you have succeeded?”, and “Have you had a definite desire to have a totally flat stomach?”. The EDE also includes items relating to the frequency and severity of binge eating episodes (e.g., “Over the past 28 days, how many times have you eaten what other people would regard as an unusually large amount of food (given the circumstances)?”). Items are rated from 0 to 6, with higher scores indicating greater frequency or severity of symptoms. The EDE is considered to be the gold-standard measurement tool for the assessment of eating disorders [24].

*The EDE-Q* [11] is the self-report questionnaire version of the gold-standard interview. The EDE-Q has been found to demonstrate good internal consistency, construct validity, and test-retest reliability [19]; Cronbach’s α = .95 for the EDE-Q global score in the present study.

*Depression Anxiety Stress Scales (DASS-21)* [25]. The DASS-21 is a 21-item self-report scale consisting of three subscales each containing 7 items that measure the severity of depression (e.g., “I tend to feel down-hearted and blue”), anxiety (“I felt I was close to panic”) and stress (e.g., “I found it difficult to relax”) symptoms occuring over the past fortnight. The DASS-21 has been found to be a valid and reliable measure with good psychometric properties e.g., [26]; Cronbach’s α = .94 for the whole measure in the present study.

*Dutch Eating Behaviour Questionnaire (DEBQ)* [17]. The DEBQ is a self-report questionnaire measuring eating behaviours and related attitudes. The DEBQ consists of three subscales: restrained eating, emotional eating and external eating; only the second two subscales were included in this study. The emotional eating scale (13 items) assesses the extent to which emotional cues trigger eating (e.g., “Do you have a desire to eat when you are irritated?”), while the external eating scale (10 items) assesses the extent to which external/environmental cues trigger eating (e.g., “If food tastes good to you, do you eat more than usual?”). Both scales were found to demonstrate adequate internal consistency [17]; Cronbach’s α = .94 for the emotional eating scale and Cronbach’s α = .79 for the external eating scale in the present study.

*Eating Disorders Beliefs Questionnaire (EDBQ)* [15]. The EDBQ is a 32-item self-report questionnaure which assesses core beliefs and underlying assumptions thought to be related to the development and maintenance of eating disorders. Participants are asked to endorse items according to how much they believe or feel them to be true from 0 (“I do not usually believe this at all”) to 100 (“I am usually completely convinced that this is true”). Higher scores indicated greater frequency and intensity of eating disordered beliefs The EDBQ contains 4 subscales: negative self-beliefs (e.g., “I’m stupid”), acceptance by others (e.g., “If my bottom is small people will take me seriously”), self-acceptance (e.g., “If my body is lean I can feel good about myself”) and control over eating (e.g., “If I binge and vomit I can stay in control”). An examination of the psychometric properties of this measure found good internal consistency, good test-retest reliability and adequate construct validity [27]. In the present study, Cronbach’s alphas ranged from .87–.96 for the subscale scores.

*Eating Disorders Core Beliefs Questionnaire (ED-CBQ)* [28]. The ED-CBQ is a 40 item self-report measure designed to measure self beliefs relevant to eating disorders. Participants rate each item for how much they believe/feel each item to be true most of the time. The ED-CBQ consists of 5 subscales: self-loathing (e.g., “putrid”), unassertiveness/inhibited (e.g., “meek”), high standards for self (e.g., “perfectionistic”), demanding/need help and support (e.g., “needy”), and abandoned/isolated (e.g., “abandoned”). Results of the initial validation study found that the ED-CBQ demonstrated adequate internal consistency and contruct validity [28]; Cronbach’s α = .92 for the whole measure in the present study.

*Difficulty in Emotion Regulation Scale (DERS)* [29]. The DERS is a 36-item self-report questionnaire that assesses difficulties in emotion regulation. The DERS has 6 subscales: non-acceptance of emotional responses (e.g., “When I am upset, I become angry with myself for feeling that way”), difficulty engaging in goal-directed behaviour (e.g., “When I am upset I have difficulty getting work done”), impulse control difficulties (e.g., “When I am upset, I become out of control”), lack of emotional awareness (e.g., “I have no idea how I am feeling”), limited access to emotional regulation strategies (e.g., “When I am upset, I believe that wallowing in it is all I can do”), and lack of emotional clarity (e.g., “I have difficulty making sense out of my feelings”). Using a scale of 1 (almost never) to 5 (almost always), participants are asked to indicate how often the items apply to themselves; higher DERS scores indicate greater emotion dysregulation. The initial validation study found the DERS subscales had good internal consistency, good test–retest reliability, and adequate construct and predictive validity [29]; Cronbach’s α = .86 for the whole measure in the present study.

### Procedure

Participants completed either the full battery of questionnaires or a brief test battery (consisting of the EBQ, the EDE-Q and the DASS-21 only), and their weight and height was recorded to determine their BMI. Participants recruited from the university completed the full test battery online, and participants recruited from the general community completed the brief test battery online. Online participants completed the questionnaires in their own time and could take breaks as required. In addition, the order of presentation of the questionnaires was randomised in the online data collection in an attempt to reduce potential fatigue effects. Clinical participants completed the brief test battery as well as completing an EDE interview administered by a doctoral level clinical psychology student who had received training in the administration of the EDE, under supervision by a senior clinical psychologist. Data for clinical participants was collected as part of ongoing treatment trials.

#### Test-retest

In total, test-retest data was collected from 63 participants (76.2% female, mean age = 27.68 years, SD = 15.68 years, mean BMI = 24.78, SD = 5.41) who completed the EBQ a second time after an interval of at least 2 weeks. Thirty-five participants from the university sample completed the EBQ a second time following an interval of two weeks (57.1% female, mean age = 19.82 years, SD = 3.5 years, mean BMI = 22.98, SD = 4.12). Sixteen treatment-seeking participants with BN who had been allocated to a waitlist condition completed the test battery a second time following an interval of six weeks (100% female, mean age = 22.31 (SD = 3.51), mean BMI = 24.16 (SD = 3.68)), twelve treatment-seeking participants with BED also allocated to waitlist condition completed the test battery a second time after an interval of ten weeks (100% female, mean age = 57.33, SD = 11.7, mean BMI = 31.15, SD = 6.4).

#### Treatment

EBQ scores were collected from two samples of individuals who participated in randomised controlled trials (RCTs) examining the efficacy of psychological treatments for binge eating. In both cases, participants were randomly allocated to either the treatment or waitlist conditions. For both RCTs, diagnosis was identified by a trained doctoral level clinical psychology student (under supervision by an experienced clinical psychologist) using the EDE semi-structured interview, and responses on self-report measures, including the EBQ, were completed at assessment (pre) and after the treatment/waitlist (post) for both groups.

#### RCT 1

Thirty-two women with a diagnosis of Bulimia Nervosa were recruited to participate in a randomised controlled trial (RCT) of a 6-week group psychological intervention as part of a research trial run at the University of Sydney. This intervention was a manualised group therapy program based on Attention Training Therapy (ATT) originally designed for the treatment of panic disorder and social phobia [30–32] . This ATT program was modified to focus on the treatment of binge eating, the program aimed to address binge eating by teaching patients to shift their attention when they experience urges to eat, and to gain the skill of thoughtful eating. Sixteen participants were randomly allocated to receive 6 weeks of group treatment for binge eating and the other sixteen participants were randomly allocated to a waitlist condition of equal duration (100% female, Mean age = 22.31 years, SD = 3.51, Mean BMI = 24.16, SD = 3.68).

#### RCT 2

Twenty-three women with a diagnosis of Binge Eating Disorder were recruited to participate in a randomised controlled trial (RCT) of a new 10-week individual psychological intervention as part of a research trial run at the University of Sydney. This intervention was a manualised individual therapy program based on Eye Movement Desensitisation and Reprocessing (EMDR) which has been found to be effective in the treatment of Posttraumatic Stress Disorder (PTSD) [33]. Eleven participants were randomly allocated to receive 10 weeks of individual treatment for binge eating (100% female, Mean age = 52.09 years, SD = 18.13 years, Mean BMI = 32.58, SD = 4.68) and the other twelve participants were randomly allocated to a waitlist condition of equal duration (100% female, Mean age = 57.33 years, SD = 11.7 years, Mean BMI = 31.15, SD = 6.4).

##### 4.4.4.1.Statistical plan

Data were cleaned prior to pooling and inspected for normality in distribution. The AMOS v12 program [34] was used to conduct a confirmatory factor analysis (CFA) on the EBQ items to evaluate the fit of the data to the hypothesised two factor model. The model was built using a Maximum Likelihood method. Aside from the CFA, all other analyses reported in this paper were conducted using the SPSS v22 program. Internal consistency was tested with Cronbach’s alpha. Reliability was tested with Pearson correlations and between group differences with Student t-test, and one-way ANOVA as appropriate.

## Results

### Confirmatory factor analysis

The data from a total of 573 participants consisting of both the community sample and the university students sample was used in the CFA analysis as it provided a homogenous non-clinical sample of adequate size, with 21 participants per item on the scale. Initially, a one-factor solution was fitted, however this solution had poor fit [35], χ^2^ = 2813.83, χ^2^ /df = 8.87, GFI = .61, Tlo = .73, CFI = .74 and RMSEA = .116 (.112–.120) This suggested that more than one factor underlies the EBQ items, which is consistent with the theory and findings of the exploratory factor analysis conducted in the development paper [10]. Therefore, the two factor model was fitted, and this solution showed an improved fit, χ^2^ = 1557.93, χ^2^ /df = 4.82, GFI = .81, Tlo = .86, CFI = .87 and RMSEA = .082 (.078–.082). However it was evident that some items were not strongly loading onto the factors and this was impacting on the goodness of fit. Hence, items were removed from the model on the basis of poor communality (less than .20), low regression weights (less than .40) and high standardised residual scores (above 2.0). Authors discussed the psychometric and theoretical value of individual items before an item was removed from the model. Following the incremental removal of these items, the resultant two-factor solution, based on16 items, demonstrated a better fit: χ^2^ = 364.15, χ^2^ /df = 3.54, GFI = .92, Tlo = .95, CFI = .96 and RMSEA = .067(.059–.074), representing an adequate-to-good fit to the data [35], refer to Table 1 for details of fit statistics for the different models. The result of an incremental model fit test suggests that the final two-factor (16-items) model provides a significantly superior fit than the original one factor model, Δχ^2^ = 2449.68, *p <* .001.

**Table 1:** Fit Statistics for the Different Models Proposed

Model	χ^2^	df	χ^2^ /df	GFI	IFI	TLI	CFI	RMSEA	AIC
1. One Factor (All 27 items)	2813.831	324	8.685	0.607	0.745	0.723	0.744	.116 (.112–.120)	2921.831
2. Two Factor (All 27 items)	1557.933	323	4.823	0.809	0.874	0.862	0.873	.082 (.078–.086)	1721.933
3. Two Factor (22 items)	912.912	208	4.389	0.862	0.915	0.905	0.914	.077 (.072–.082)	1002.912
4. Two Factor (21 items)	765.081	188	4.07	0.878	0.926	0.917	0.926	.073 (.068–.079)	851.081
5. Two Factor (19 items)	651.478	151	4.314	0.886	0.930	0.921	0.930	.076 (.70–.82)	729.478
6. Two Factor (16 items)	364.15	103	3.535	0.923	0.956	0.949	0.956	0.067 (.059–.074)	430.15

The factor structure of the final two-factor model of the EBQ is presented in Table 2. Factor 1 (labelled as “Negative Beliefs”) is defined by the items capturing those negative (or no control) metacognitive beliefs about eating and food. Factor 2 (labelled as “Positive Beliefs”) is defined by the items capturing those positive metacognitive beliefs about eating and food. Positive and negative subscales correlated positively with each other, *r* = .63, suggesting the possibility of a higher-order factor.

**Table 2:** Results of a Confirmatory Factor Analysis of the EBQ (*N* = 573) Standardised Regression Weights & Communality

EBQ Items	F1: Negative Beliefs	F2: Positive Beliefs	Communality (h2)
I will never be able to control my urges to eat	.656		.431
My eating will always need to be controlled	.626		.392
Once I start eating I can’t stop	.796		.633
I have no willpower in relation to food	.738		.544
I can’t control my eating because I am weak	.829		.687
If I don’t control myself I would never stop eating	.770		.593
There is nothing I can do to stop eating	.671		.451
Eating helps me to cope		.673	.453
Eating helps to reduce unpleasant physical feelings		.646	.417
Eating means I don’t have to think about negative things		.781	.610
Eating helps to control my emotions		.774	.599
Eating keeps my feelings at a tolerable level		.762	.580
Eating helps me to cope with negative thoughts		.897	.805
Eating helps me to cope with unpleasant physical sensations		.750	.562
Eating helps me cope with negative feelings		.893	.797
Eating helps to stop feelings that I don’t like		.870	.758
Correlation (F1 & F2)	*r* = .63		

### Psychometric evaluation of the two-factor EBQ

Based on the 16-item EBQ that resulted from the CFA, the psychometric properties of the measure, and its two subscales Negative Beliefs (NBS) and Positive Beliefs (PBS), were assessed.

Table 3 summarises the mean total scores and subscale scores for different sample groups and subgroups of participants. As expected, clinical participants (*n =* 196*)* scored significantly higher on the Total EBQ and EBQ subscales than participants recruited from the non-clinical (*n =* 573) sites (Total EBQ: clinical sample mean = 52.32, SD = 12.91, non-clinical sample mean = 36.05, SD = 13.00; *F* (1767) = 229.41, *p < .01,* η_p_^2^ *=* .23; NBS: clinical sample mean = 23.75, SD = 6.19, non-clinical sample mean = 15.08, SD = 6.05; *F* (1767) = 296.79, *p < .01,* η_p_^2^ *=* .28; PBS: clinical sample mean = 28.57, SD = 9.08, non-clinical sample mean = 20.97, SD = 8.50; *F* (1767) = 112.516, *p < .01,* η_p_^2^ *=* .13). Results from one-way ANOVAs found significant differences, with moderate-to-large effect sizes (partial eta squared (η_p_^2^) > 0.10), between EBQ scores for participants who self-reported to be engaging in a clinical level of binge eating (at least three binge episodes accompanied by a sense of loss of control in the past month as reported in the EDE-Q) compared to participants who reported that they did not engage in any binge episodes in the previous month,^1^ EBQ Total Score: *F(1594.065)* = 177.81, *p < .01,* η_p_^2^  *=* .22*,* NBS: *F(1589.677)* = 180.32, *p < .01,* η_p_^2^  *=* .22*,* PBS: *F(1568.120)* = 112.10, *p < .01,* η_p_^2^  *=* .15.

**Table 3:** EBQ Subscale Group Performance

	Total EBQ Score Mean (SD)	Negative Beliefs Mean (SD)	Positive Beliefs Mean (SD)
Sample Groups			
Community Sample (*n* = 290)	37.68 (13.53)	15.86 (6.50)	21.82 (8.85)
University Sample (*n* = 283)	34.39 (12.24)	14.28 (5.44)	20.10 (8.06)
Binge Eating Research Sample (*n* = 76)	54.32 (13.31)	24.49 (6.34)	29.83 (9.56)
Obesity Research Sample (*n* = 120)	51.02 (12.54)	23.28 (6.07)	27.77 (8.70)
Subgroups			
Non Binge Eating (*n* = 249)	32.39 (12.30)	13.47 (5.60)	18.92 (8.12)
Engaging in Binge Eating (*n* = 347)	46.95 (14.25)	20.51 (7.17)	26.45 (9.16)
BMI in Normal Range (*n* = 419)	37.10 (13.93)	15.60 (6.65)	21.50 (8.74)
BMI >25 (*n* = 297)	45.43 (14.88)	20.15 (7.04)	25.28 (9.31)

Significant differences were also found for EBQ scores between participants with a BMI in the normal range compared to participants with a BMI >25, with small-to-moderate effect sizes (η_p_^2^ < 0.10); EBQ Total Score: *F(1714)* = 58.96, *p < .01,* η_p_^2^  *=* .08*;* NBS: *F(1714)* = 77.42, *p < .01,* η_p_^2^  *=* .10*;* PBS^1^: *F(1579.316)* = 28.86, *p < .01,* η_p_^2^  *=* .04.

### Internal consistency

Cronbach’s alphas were calculated with the full sample (*N* = 769), as well as the different sample groups, refer to Table 4 below. EBQ subscales were found to have good internal consistency with Cronbach’s alphas between .84 and .93 for the EBQ Total Score, Cronbach’s alphas between .76 and .91 for the NBS and Cronbach’s alphas of .92 and .94 for the PBS.

**Table 4:** Internal Consistency of EBQ Scales across Different Sample Groups

	EBQ Total	EBQ NBS	EBQ PBS
Full Sample (*N* = 769)	α = .94	α = .91	α = .94
Sample Groups			
Community Sample (*n* = 290)	*α = .*93	*α =* .89	*α* = .94
University Sample (*n* = 283)	*α* = .93	*α =* .87	*α* = .93
Binge Eating Research Sample (*n* = 76)	*α =* .84	*α = .*76	*α* = .92
Obesity Research Sample (*n* = 120)	*α =* .92	*α =* .86	*α* = .94

### Reliability

Test-retest reliability was calculated for the EBQ positive and negative belief subscales across the three samples that completed the EBQ a second time after an interval of 2–10 weeks (university student participants were tested again after 2 weeks, the waitlist group from the BN group treatment trial were tested again after an interval of 6 weeks, and the waitlist group from the BED treatment trial were tested again after an interval of 10 weeks). The results showed excellent test-retest reliability: paired samples *t-*tests found no significant difference between Time 1 and Time 2 scores (Total, NBS or PBS) across the three samples: university students, BN patients, or BED patients, refer to Table 5. In addition, Pearson’s *r* correlations between Time 1 and Time 2 scores were significant at *p* < .001, Total, *r* = .91*,* NBS, *r =* .85 or PBS, *r =* .84.

**Table 5:** Test-retest reliability. Results of t-test for Time 1 and Time 2 scores across three samples

	EBQ Total Score Mean (SD)				Negative Beliefs Mean (SD)				Positive Beliefs Mean (SD)			
	T1	T2	t	*p*	T1	T2	t	*p*	T1	T2	t	*p*
Students (2wks) *n* = 35	35.83 (14.18)	35.00 (13.24)	.76	.45	15.66 (6.55)	15.57 (6.58)	.17	.87	20.17 (8.61)	19.43 (8.04)	.95	.35
BN (6wks) *n* = 16	52.44 (10.66)	52.31 (8.37)	.06	.95	24.44 (5.29)	24.19 (5.69)	.16	.87	28.00 (7.80)	28.13 (5.82)	−.10	.92
BED (10wks) *n* = 12	57.92 (11.02)	59.41 (9.31)	−.82	.43	25.41 (5.21)	24.67 (4.85)	.72	.49	32.50 (9.69)	34.75 (6.36)	−1.01	.33

*T1* Time 1 / Pre scores, *T2* Time 2 / Post scores

### Construct validity

Convergent validity was calculated by assessing correlations between EBQ subscale scores and measures of specific eating disorder symptoms and related psychopathology, refer to Table 6. Significant correlations were identified between the EBQ total and subscale scores and all included measures of related behaviour and psychopathology, most of these correlations are positive and fall in the moderate range (notable exceptions identified in italics or bold in Table 5, refer to Discussion).

**Table 6:** Correlations between EBQ Scores and Measures of Eating-Related and General Psychopathology

	EBQ Total Score	Negative Beliefs Scale	Positive Beliefs Scale
*n = 767*			
BMI	.334^a^	.362^a^	.253^a^
*n = 648*			
DASS-21 Depression	.503^a^	.483^a^	.429^a^
DASS-21 Anxiety	.418^a^	.361^a^	.387^a^
DASS-21 Stress	.457^a^	.394^a^	.425^a^
*n = 610*			
EDE-Q - Objective Binge Episodes (OBE)	.389^a^	.395^a^	.316^a^
EDE-Q – OBE + Loss of Control	.515^a^	.555^a^	.391^a^
EDE-Q Global Score	.643^a^	**.726** ^a^	.464^a^
EDE-Q Restraint	.388^a^	.517^a^	.221^a^
EDE-Q Eating Concern	.643^a^	**.720** ^a^	.469^a^
EDE-Q Shape Concern	.630^a^	.683^a^	.477^a^
EDE-Q Weight Concern	.647^a^	.694^a^	.494^a^
*n = 283*			
EDBQ Negative Self-Beliefs	.542^a^	.541^a^	.458^a^
EDBQ Acceptance by Others	.563^a^	.581^a^	.463^a^
EDBQ Self-Acceptance	.423^a^	.486^a^	.313^a^
EDBQ Control over Eating	.564^a^	.644^a^	.422^a^
ED-CBQ Self-Loathing	.493^a^	.474^a^	.429^a^
ED-CBQ Demanding/Needing Help	.494^a^	.466^a^	.436^a^
ED-CBQ Unassertive/Inhibited	.460^a^	.421^a^	.414^a^
ED-CBQ High Standards for Self	*−.146* ^*b*^	*−.172* ^*b*^	*−.106* ^*b*^
DERS Total Score	.562^a^	.521^a^	.501^a^
DEBQ Emotional Eating	.674^a^	.611^a^	.610^a^
DEBQ External Eating	.371^a^	.361^a^	.320^a^

^a^Pearson Correlation is significant at the 0.01 level (two-tailed)

^b^Pearson Correlation is significant at the 0.05 level (two-tailed)

## Responsiveness – Sensitivity to treatment

### Treatment group 1: Six week group psychological intervention for BN

The scores of the 16 participants who completed the treatment program were compared to those pre and post scores of 16 treatment-seeking participants with BN who were allocated to a waitlist for a period of six weeks. Table 7 summarises the group means, SDs and Cohen’s *d* effect sizes at pre and post time-points for both the treatment and waitlist group.

**Table 7:** EBQ scores at pre and post for treatment and waitlist groups

	EBQ Total Score Mean (SD)			Negative Beliefs Mean (SD)			Positive Beliefs Mean (SD)		
	Pre	Post	d	Pre	Post	d	Pre	Post	d
BN Treatment (6 weeks, *n* = 16)	59.13 (6.79)	51.44 (9.51)	.93	27.31 (2.98)	21.38 (6.52)	1.17	31.81 (5.83)	30.06 (7.00)	.27
BN Waitlist (6 weeks, *n* = 16)	52.44 (10.66)	52.31 (8.37)	.01	24.44 (5.29)	24.19 (5.69)	.05	28.00 (7.80)	28.13 (5.82)	.02
BED Treatment (10 weeks, *n* = 11)	54.45 (10.03)	40.00 (10.50)	1.41	24.82 (3.57)	17.82 (5.11)	1.59	29.64 (9.01)	22.18 (6.85)	.93
BED Waitlist (10 weeks, *n* = 12)	58.58 (10.81)	59.41 (9.31)	.08	25.42 (5.21)	24.67 (4.85)	.15	33.17 (9.24)	34.75 (6.36)	.20

The EBQ total scores as well as the subscale scores were analysed by mixed design 2 x (2) ANOVA with a between-subject factor of Condition (treatment vs waitlist) and a within-subject factor of Time (pre vs post). For the EBQ Total score and NBS, significant main effects of both Condition and Time were observed (all *p*s < .05). Additionally, the EBQ Total Score and the NBS showed significant interaction effects (time x condition), EBQ Total: *F*(1,30) = 5.011, *p* = .033, η_p_^2^  = .143, NBS: *F*(1,30) = 6.693, *p* = .015, η_p_^2^ = .182. For both the EBQ Total score and the NBS, analyses of the simple main effects found that these interaction effects were driven by significant differences were between pre and post scores for participants allocated to the treatment condition, whereby post scores were significantly lower than pre scores, EBQ Total: *F*(1,30) = 10.356, *p* = .003, η_p_^2^  = .257; NBS: *F*(1,30) = 14.589, *p* = .001, η_p_^2^  = .327. No significant differences were found between pre and post scores for participants allocated to the waitlist condition, EBQ Total: *F*(1,30) = .003, *p* = .959, η_p_^2^  < .001; NBS: *F*(1,30) = .026, *p* = .873, η_p_^2^  = .001. Although there were no significant main effects or interactions found for the PBS for this sample, the observed power for the comparisons for the PBS was low, so inferences cannot be drawn about the effect in this case.^2^

### Treatment group 2: 10 week individual psychological intervention for BED

The scores of the 11 participants who completed the treatment program were compared to those pre and post scores of 12 treatment-seeking participants with BED allocated to waitlist for a period of ten weeks. Table 7 summarises the group means, SDs and Cohen’s *d* effect size at pre and post time-points for both the treatment and waitlist groups.

Again, the EBQ total score as well as the subscale scores were analysed by mixed design 2 x (2) ANOVA with a between-subject factor of Treatment Condition (treatment vs waitlist) and a within-subject factor Time (pre vs post). Significant main effects of Condition were observed for EBQ Total, NBS and PBS (all *ps* < .05). Significant main effects of Time were observed for EBQ Total and NBS (*p*s < .01), but no significant main effect of Time was found for the PBS (*p* = .052). The EBQ Total Score, NBS and the PBS showed significant interaction effects (time vs. condition), EBQ Total: *F*(1,21) = 18.85, *p* < .01, η_p_^2^ = .47, NBS: *F*(1,21) = 10.32, *p* < .01, η_p_^2^ = .33, PBS: *F*(1,21) = 10.09, *p* < .01, η_p_^2^ = .33. In all three cases (EBQ Total, NBS and PBS), analyses of the simple main effects found that these interaction effects were driven by significant differences between pre and post scores for participants allocated to the treatment condition, whereby post scores were significantly lower than pre scores, EBQ Total: *F*(1,21) = 32.30, *p* < .001, η_p_^2^ = .61; NBS: *F*(1,21) = 24.81, *p* < .001, η_p_^2^  = .54; PBS: *F*(1,21) = 13.16, *p* = .002, η_p_^2^  = .39, but no significant differences between pre and post scores for participants allocated to the waitlist condition, EBQ Total: *F*(1,21) = .12, *p* = .74, η_p_^2^ = .01; NBS: *F*(1,21) = .31, *p* = .58, η_p_^2^ = .02; PBS: *F*(1,21) = .65, *p* = .43, η_p_^2^ = .030.^3^

## Discussion

The purpose of this study was to examine the validity of the two-factor model of the Eating Beliefs Questionnaire (EBQ) [10] by conducting a CFA with a large homogenous sample. This study also aimed to assess the psychometric properties, validity and reliability of the EBQ using both a clinical sample of treatment-seeking patients with BN or BED, and a non-clinical sample.

The results of the CFA provided support for the two-factor model proposed by Groves et al. [10] in the development paper; a two-factor model (negative beliefs and positive beliefs) was found to provide a better fit to the data than a one-factor solution. Following an assessment of the psychometric and theoretical value of the 27 original items, the authors agreed to remove 11 items that had demonstrated relatively poor psychometric value with either low communality, low regression weight or high standardised residual weight. This resulted in a 16 item scale (7 items loading onto the negative beliefs factor and 9 items loading onto the positive beliefs factor). The two-factor solution was found to provide adequate-to-good fit to the 16-item scale, representing an improvement in fit for the shorter version of the questionnaire compared to the original 27-item scale.

The psychometric properties of the 16-item EBQ, and its two subscales, were assessed. Overall, the EBQ was found to be a valid and reliable measure with evidence for its psychometric properties. The total EBQ and its subscale scores were found to have good internal consistency across the different sample groups. Furthermore, the EBQ and subscales showed good test-retest reliability in both a clinical and non-clinical sample, across intervals of 2 to 10 weeks.

Good convergent validity was evidenced by the significant correlations between the EBQ scores and relevant measures of eating disordered and related psychopathology, including BMI (*r =* .25 to .36), number of binge episodes, EDE-Q subscales, DASS-21 subscales, eating disorder measures (EDBQ, ED-CBQ and DEBQ), and a measure of poor emotional regulation (DERS). Of particular interest is the strong positive correlations (*r* > .70, bolded in Table 5) found between the EBQ Negative Beliefs Subscale (NBS) and EDE-Q Global Score and EDE-Q Eating Concern Subscale. As well as the negative correlations found between EBQ scores and the ED-CBQ High Standards for Self Subscale score (italicised in Table 5). These findings are consistent with the literature as the ED-CBQ High Standards for Self Subscale identifies positive beliefs about the self (e.g., “I am conscientious”), whereas binge eating or general eating disordered behaviour is thought to be associated with poor self-esteem [36, 37], this is supported by the significant positive correlations identified between EBQ scores and ED-CBQ Self-loathing subscale, and the ED-CBQ Negative Self Beliefs subscale. These findings not only demonstrate the convergent validity of the EBQ scales, but also provide evidence for the relevance of the metacognitive beliefs being measured by the EBQ – positive and negative beliefs about food and eating. These results indicate that these two types of metacognitive beliefs are related to eating disorder symptomatology (EDE-Q), increased BMI, measures of other beliefs relevant to eating disorders (EDBQ, ED-CBQ, & DEBQ) and a measure of emotion dysregulation (DERS). However, it is important to note that although the order of questionnaire presentation was randomised to reduce the potential impact of testing fatigue, the participants completing the full test battery may have experienced fatigue effects.

Scores on the EBQ and its subscales were found to differentiate between subgroups of participants. EBQ scores were significantly higher for participants recruited from clinical samples (BN, BED and obesity studies) compared to scores of participants recruited from non-clinical sites (university and general community). EBQ scores were significantly higher for participants who self-reported regular binge eating compared to scores of participants who reported no presence of binge eating. In addition, EBQ scores were significantly higher for participants who had a BMI in the overweight range or greater compared to scores of participants with a BMI in the normal range. It most likely that the relationship between higher BMI and elevated EBQ scores is driven by the high co-morbidity of increased body-weight and difficulties with problematic eating behaviour, including binge eating [3, 4].

EBQ scores were also found to be responsive to psychological treatment in two different RCT treatment studies. EBQ total scores were found to be significantly lower at post-treatment for participants who received treatment compared to participants allocated to waitlist. Participants with a diagnosis of BN allocated to a 6 week group ATT therapy treatment showed a significant reduction in EBQ total score and NBS scores, but not PBS scores. Participants with a diagnosis of BED allocated to a 10 week individual EMDR-based therapy treatment showed a significant reduction in scores across EBQ total, NBS and PBS subscales. Differences in the results between these two trials can be explained by the differences in the treatment modality (ATT or EMDR based therapies), delivery (group or individual) and dose (6 or 10 weeks), as well as pre-existing differences between the patients involved in the two different trials (BN compared to BED). Effect sizes were larger for the EMDR treatment trial with BED. This is likely because patients allocated to treatment in the BED trial received 10 weeks of individual treatment. Therefore, these patients received a higher dose of treatment in terms of the number of sessions (10 versus 6), but also due to the nature of the delivery of the treatment as they had individual sessions compared to patients in the BN trial who received 6 weeks of group treatment. Although the there was no significant difference identified between pre and post scores on the PBS for those in the BN group ATT therapy trial, the observed power for the comparisons for the PBS was low, so inferences cannot be drawn about the effect in this case. However, a possible explanation for why the effect size was smaller for the PBS than for the NBS is that the ATT treatment used in this trial targeted the patients’ sense of loss of control over urges to binge eat, but did not target positive beliefs about eating. Indeed, across both trials, the change between pre and post treatment scores was greater for the NBS than the PBS. This finding may indicate that negative beliefs are easier to shift in treatment than positive beliefs are, or perhaps that treatments are usually geared towards building up the client’s sense of control over their eating, but neglect to address their beliefs that eating/food helps them to cope. Future studies should examine whether treatments that also address the positive beliefs about eating lead to better long-term outcomes.

One limitation of the current study is the relatively small size of the treatment-seeking eating disordered sample that was used to assess the test-retest reliability and sensitivity to treatment of this measure. As a result, our knowledge on the utility of this measure with eating disorder patients remains limited. For example, we were unable to calculate group norms for patients with a diagnosis of BN versus BED. We were also limited by the fact that the psychological interventions being administered in the two RCTs described in this paper were novel treatments, with no comparison with an evidence-based treatment such as Enhanced Cognitive Behavioural Therapy (CBT-E) [38]. Therefore, future studies should examine the validity of this measure in a large treatment-seeking eating disordered sample to provide clinically useful normative data, and also assess the EBQ’s sensitivity to treatment in the context of an evidence-based CBT treatment program for binge eating.

A further limitation is that the EBQ does not provide a measure of the third type of beliefs that Cooper, Wells and Todd [8] identified in their model of BN as playing an important role in perpetuating binge eating behaviour, permissive beliefs about eating. In order for the EBQ to provide a thorough assessment of the three types of beliefs hypothesised by Cooper et al. to be important to the maintenance of binge eating behaviour, it is recommended that future research develop additional items for the EBQ that assess permissive beliefs about binge eating [8].

However, as this study used a large and diverse sample, this paper offers a thorough assessment of the psychometric properties of the EBQ, including test-retest reliability and sensitivity to treatment, across both the general community, but also in a treatment-seeking eating disordered clinical sample. Moreover, the number and variety of relevant measures included in the test-battery allowed us to assess the construct validity of the EBQ, not only against measures of eating disordered behaviours and body mass index, but also with other measures of cognitions and beliefs that have been hypothesised to be relevant to the development and perpetuation of binge eating behaviour such as poor emotional regulation [36, 37], negative beliefs about the self [8, 39], and body shape concerns [40].

## Conclusion

The present study validates an existing measure of positive and negative metacognitive beliefs about eating with a clinical and non-clinical sample, providing valuable information about the utility of the EBQ as a measure for use in both clinical and research settings. This measure is unique and one of only a few cognitive measures that specifically addresses beliefs relevant to binge eating, a behaviour common to BN, atypical eating disorders and, more specifically, BED patients. The EBQ provides a valuable tool for assessing and measuring change in key maintaining cognitions during the treatment of binge eating. Clinicians can also use the EBQ as tool to help inform formulation and treatment planning for clients seeking treatment for binge eating; clinicians can observe which items on the EBQ clients endorse most strongly and use these responses to plan an individualised and targeted treatment program to address problematic beliefs about food and binge eating held by their client.

Overall, these findings indicate that the EBQ is a psychometrically sound self-report measure that can be used to assess the positive and negative beliefs about eating that are thought to contribute to the maintenance of binge eating behaviour in eating disordered individuals. The EBQ provides a reliable cognitive measure that has been found to be sensitive to psychological treatment, suggesting that the EBQ could be particularly useful in assessing the outcome of cognitive therapy, or the impact of any intervention, on the underlying metacognitive beliefs about positively and negatively perceived aspects of binge eating.

## Abbreviations

AIC: Akaike Information Criterion
AN: Anorexia Nervosa
ANOVA: Analysis of Variance
ATT: Attention Training Therapy
BED: Binge Eating Disorder
BES: Binge Eating Scale
BITE: Bulimic Investigatory Test Edinburgh
BMI: Body Mass Index
BN: Bulimia Nervosa
BULIT-R: Bulimia Test Revised
CBT: Cognitive Behavioural Therapy
CBT-E: Enhances Cognitive Behavioural Therapy
CFA: Confirmatory Factor Analysis
CFI: Comparative Fit Index
DASS-21: Depression Anxiety Stress Scales
DEBQ: Dutch Eating Behaviour Questionnaire
DERS: Difficulty in Emotion Regulation Scale
df: degrees of freedom
EAT-26: Eating Attitudes Test
EBQ: Eating Beliefs Questionnaire
EDBQ: Eating Disorders Beliefs Questionnaire
ED-CBQ: Eating Disorders Core Beliefs Questionnaire
EDDS: Eating Disorders Diagnostic Scale
EDE: Eating Disorders Examination
EDE-Q: Eating Disorders Examination Questionnaire
EMDR: Eye Movement Desensitisation and Reprocessing
GFI: Goodness of Fit Index
NBS: Negative Beliefs Scale
OBE: Objective Binge Eating Episodes
PBS: Positive Beliefs Scale
PTSD: Posttraumatic Stress Disorder
RCT: Randomised Controlled Trial
RMSEA: Root Mean Square Error of Approximation
SD: Standard Deviation
SPSS: Statistical Package for Social Sciences
TFEQ: Three Factor Eating Questionnaire
Tlo: Tucker-Lewis Index

## Footnotes

1 Unequal variances were observed for the responses between non binge eating and binge eating groups, and between the normal BMI and higher BMI groups for the PBS only (Levene’s Test of Equality of Error Variances, all *ps* < .05). As the assumptions of homogeneity of variance were not met, the results from Welch Robust Test of Equality of Means have been used.

2 Observed power computed using alpha = .05. In this sample, observed power for EBQ Total = .61, EBQ Total*Time = .58; observed power for EBQ NBS = .77, EBQ NBS*Time = .71; observed power for EBQ PBS = .12, EBQ PBS*Time = .14.

3 Observed power computed using alpha = .05. In this sample, observed power for EBQ Total = .96, EBQ Total*Time = .99; observed power for EBQ NBS = .97, EBQ NBS*Time = .87; observed power for EBQ PBS = .50, EBQ PBS*Time = .86.
